# Machinability of INCONEL718 Alloy with a Porous Microstructure Produced by Laser Melting Powder Bed Fusion at Higher Energy Densities

**DOI:** 10.3390/ma13245730

**Published:** 2020-12-15

**Authors:** Paul Wood, Antonio Díaz-Álvarez, José Díaz-Álvarez, María Henar Miguélez, Alexis Rusinek, Urvashi F. Gunputh, Gavin Williams, Slim Bahi, Judyta Sienkiewicz, Paweł Płatek

**Affiliations:** 1 Institute of Innovation in Sustainable Engineering (IISE) University of Derby, Quaker Way, Derby DE1 3HD, UK; p.wood7@derby.ac.uk (P.W.); u.gunputh@derby.ac.uk (U.F.G.); G.Williams1@derby.ac.uk (G.W.); 2 Department of Mechanical Engineering, University Carlos III of Madrid, Avda. Universidad 30, 28911 Madrid, Spain; andiaza@ing.uc3m.es (A.D.-Á.); mhmiguel@ing.uc3m.es (M.H.M.); alexis.rusinek@univ-lorraine.fr (A.R.); 3 Laboratory of Microstructure Studies and Mechanics of Materials (LEM3), Lorraine University, UMR CNRS 7239, 57078 Metz, France; slim.bahi@gmail.com; 4 Faculty of Mechatronics, Armament and Aerospace, Military University of Technology, gen. Sylwestra Kaliskiego 2, 00-908 Warsaw, Poland; judyta.sienkiewicz@wat.edu.pl (J.S.); pawel.platek@wat.edu.pl (P.P.)

**Keywords:** SLM, additive manufacturing, spherical porosity, machining, powder bed fusion, INCONEL718

## Abstract

Products produced by additive manufacturing (AM) seek to exploit net shape manufacturing by eliminating or minimizing post-process stages such as machining. However, many applications which include turbo machinery components with tight dimensional tolerances and a smooth surface finish will require at least a light machine finishing stage. This paper investigates the machinability of the additively fabricated INCONEL718 (IN718) alloy produced by laser melting powder bed fusion (LM-PBF) with different levels of spherical porosity in the microstructure. The literature suggests that the band width for laser energy density, which combines the various scan process parameters to obtain a low spherical type porosity in the LM-PBF IN718 alloy (~1%), has wide breadth. With the increasing laser energy density and above a threshold, there is a rapid increase in the spherical pore size. In this paper, three tube samples each with different levels of spherical porosity were fabricated by varying the laser energy density for LM-PBF of the IN718 alloy within the stable and higher energy density range and the porosity measured. A low laser energy density was avoided due to balling up, which promotes highly irregular lack of fusion defects and poor consolidation within the alloy microstructure. An orthogonal turning test instrumented, with a three-component dynamometer to measure the cutting forces, was performed on AM produced IN718 tube samples under light cut conditions to simulate a finish machining process. The orthogonal turning tests were also performed on a tube sample obtained from the wrought extruded stock. The machining process parameters, which were studied include varying the cutting speed at three levels, at a fixed feed and under dry cut conditions for a short duration to avoid the tool wear. The results obtained were discussed and a notable finding was the higher rate of built-up-edge formation on the tool tip from the AM samples with a higher porosity and especially at a higher cutting speed. The paper also discusses the mechanisms that underpin the findings.

## 1. Introduction

**Figure 1:**
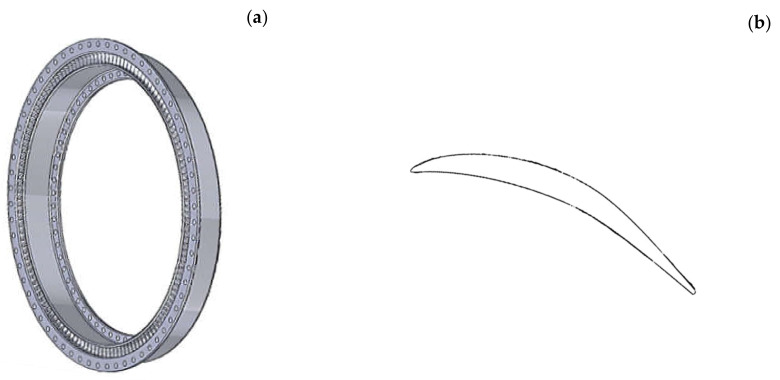
(**a**) Outlet guide vane 605 mm outside diameter; (**b**) mid-section geometry of NACA-6 series airfoil of the outlet guide vane.

Nickel-based superalloys such as INCONEL718 (IN718) are used in applications such as jet engines that require high resistance to fatigue and corrosion at high temperatures. Nickel-based alloys account for between 30% and 40% [1,2] of the weight of a large turbo fan engine and IN718 accounts for more than 30% of all the super alloy produced [3]. The geometry of most engine components is complex. An outlet guide vane (OGV) in the last stage of a compressor is displayed in Figure 1 measuring 605 mm in outer diameter with 140 equally spaced vanes. Separating each vane is an airfoil and manufacturing precision [4] for the leading and trailing edge thickness may typically not exceed +/−0.06 mm for a NACA-6 series type airfoil profile. Manufacturing an OGV component involves multistage forging with extensive machining to achieve the final shape within the tolerances required. Machining processes are a significant cost in engine manufacturing and estimates [1] suggest it could be as high as 35% of the component cost.

The tool wear rate in machining nickel-based superalloys is high [5,6,7] and cutting speed (Vc), feed rate (f), depth of cut (DoC) or width of cut (WoC), and tool paths require careful management to avoid the tensile residual stress developing at the surface of the component [4,8,9]. The IN718 alloy has a low thermal conductivity and high work hardening rate. To prevent the high temperature from developing at the chip, tool, and workpiece surface, cutting parameters Vc (m/min), f (mm/rev), and DoC (mm) are limited to ranges, respectively for finishing (or light cutting) Vc = 30 to 60, f = 0.07 to 0.25, and DoC = 0.3 to 1.5. Increasing Vc in turning the IN718 alloy from 46 to 76 m/min [5] displayed a significant increase in the tool wear for a range of physical vapour deposition (PVD) coated carbide tools, achieving between 20% and 30% of the tool life at the higher cutting speed. More recent works [10] by measuring cutting forces in finish turning the IN718 alloy with the coated carbide tooling suggests similar reductions of 20% to 30% in the tool life by increasing Vc from 35 to 50 m/min with f = 0.1 mm/rev. The feasibility of dry cut finish turning of the IN718 alloy was studied [11] with Vc in the range from 50 to 70 m/min by examining tip wear patterns and the force measurement with various inserts. The feasibility of the dry cut turning was demonstrated for a carbide coated tip with a zero rake angle over a small length of 0.17 mm, and although the surface roughness increased slightly, the tool life was within reasonable bounds. Other published works [8,12,13,14,15] covering milling and turning of the IN718 alloy, comparing dry cut, conventional coolant systems, cryogenic and the minimum quantity lubricant (MQL) had sought to determine the productivity improvement for roughing and finishing. 

The temperature measured [16] at the tool-chip interface increases with Vc, at 30 and 90 m/min, respectively, and was 910 and 1130 °C and [17] observed the same trend and range. The chip temperature measured by the infrared camera [6,7] in turning the IN718 alloy with MQL and Vc = 60 m/min was 575 °C, and under dry cut the chip temperature increased to 846 °C. A fiber-optic two-color pyrometer [15] was used for the localized workpiece temperature measurement in turning the IN718 alloy in the range Vc = 60 to 180 m/min and the workpiece surface temperature increased slightly from 420 to 450 °C with f = 0.05 mm/rev at higher Vc, but decreased slightly from 295 to 255 °C at higher Vc with f = 0.1 mm/rev. As temperature increases in the localized zone of plastic deformation a tendency for strain softening (shear band formation) develops, creating a severely serrated chip form [7] resulting in a roughened surface and increased residual tensile stress on the surface of the workpiece. Using X-ray diffraction, the surface residual tensile stress [8] in turning the IN718 alloy with Vc = 60 m/min, f = 0.05 mm/rev, and DoC = 0.63 mm under lubricated and dry conditions was in the range from 650 to 780 MPa. A transition cutting speed at which the chip form changes from a continuous to a serrated chip for the IN718 alloy was found [7,18] to be approximately 61 m/min.

The adoption of additive manufacturing (AM) by the industry [19,20] is accelerating as a rapid fabrication method for metal components. Among the AM fabrication methods, laser melting powder bed fusion (LM-PBF) is the most versatile only limited by an individual part size that can be produced typically within a build space of up to 400 × 400 × 400 mm. The LM-PBF of turbomachinery components in nickel-based alloys has been used to fabricate blades for the industrial power plant [20]. Larger components such as an OGV, which is a static (nonrotating) component could be fabricated in smaller segments [21] and then assembled. The LM-PBF can reduce the waste streams associated with conventional fabrication processes [19], thereby requiring fewer fixtures, cutting tools, less swarf, and metal working fluid which lower greenhouse gas emissions. 

The LM-PBF uses a laser beam (either continuous or pulsed) to fully melt a thin layer of metal powder typically up to 100 μm thickness in an inert gas atmosphere. After rapid solidification, a new layer of powder is deposited, and the laser scans the deposited powder layer, fusing it to the substrate (the part section to be produced) following a specific scan path. The process is repeated layer by layer until the geometry of the part is obtained. The process melt pool dynamics involves the generation of high temperatures with fast cooling rates and highly localised thermal gradients that create a unique microstructure in the alloy, which can enhance mechanical properties [22,23,24,25,26,27,28,29,30,31].

The LM-PBF process parameters include laser power (P), scan velocity (V), layer height (D), hatch spacing (H), laser spot size (d), laser scanning method (raster, chessboard), powder particle size (distribution and sphericity), etc. To a first approximation, the volume energy density (VED) is used to select the correct combination of process parameters to design the LM-PBF process [32] for the part to be produced:

VED = P/(V.H.D)(1)

Although dependent on the part build orientation on the build plate and specific process parameters chosen, e.g., P, V, D, and H, the microstructure of the LM-PBF IN718 alloy has generally been observed to have directionality [31] with columnar grains in the build axis direction, and smaller equiaxed grains when viewed on the build axis plane. This results in directionally dependent mechanical properties associated with the build orientation. Studies have been published [30] to establish the optimum combination of process parameters for LM-PBF metals and alloys such as 316 L, Ti-6Al-4 V, and AlSi10 Mg, seeking to minimize process defects and surface roughness, improve alloy integrity, and increase process productivity. More recently, the IN718 alloy has received increased interest [13,28,31,33]. Despite the capabilities of the LM-PBF and the refinements reported, it can only be considered a near net shape manufacturing process for high precision components. To date, only one published work [6] studied the machinability of LM-PBF produced IN718 alloy using process parameters (P = 200 W, H = 90 micron, D = 60 micron, V = 875 mm/s) by turning a 16 mm diameter bar with Vc = 60 m/min, DoC = 0.4 mm, and f (mm/rev) = 0.08, 0.16, and 0.2. The measures examined were the surface integrity, surface roughness, microhardness, and XRD. 

A disadvantage often cited for LM-PBF alloys is the porosity and defects that develop in the alloy microstructure [25,26,28]. However, these can be minimized by the selection of specific process parameters together with post-processing treatments such as a hot isostatic pressing (HIP) which can eliminate most types of porosity below a certain size [32]. It may be preferable to machine the alloy before applying a post-process HIP treatment followed by solution and aging treatments to bring the alloy to full strength. Porosity defects may be grouped according to the VED regime in which they lie. With energy density too low, lack of fusion (LoF) type defects developed due to balling up [30]. This promotes highly irregular lack of fusion type defects and an alloy microstructure with diminished structural integrity. With the increasing laser energy density and above a threshold, there is a rapid increase in the spherical pore size. The literature suggests that the band width for VED to obtain a spherical porosity below 1% in the LM-PBF IN718 alloy is broad.

The paper investigates the machinability of the AM IN718 alloy produced by LM-PBF with different levels of spherical porosity in the microstructure. The applied investigation methodology takes the following steps:

1. Fabrication of AM IN718 tube samples with different levels of spherical porosity by varying the laser energy density within the stable and higher energy density range.
2. Instrumented machining to measure cutting forces in the orthogonal turning of tube samples performed under light cut conditions to simulate a finishing process. The feed motion is along the length of the tube axis and the width of cut across the full tube thickness, performed under dry cut (no coolant) conditions.
3. Microhardness and porosity analysis of the AM tube samples on a plane perpendicular to the direction of the feed motion of the cutting tool.
4. Machining of the same alloy grade produced as a wrought (extruded) tube product to compare the machinability of the alloy produced by the two fabrication methods.

## 2. Experimental Preparations for Additive Manufacturing and Machining

### 2.1. Methods and Materials

**Figure 2:**
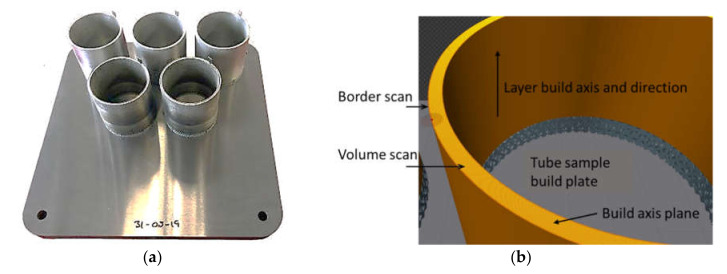
Additive manufacturing (AM) fabricated tube samples in the Renishaw AM250 SLM machine (**a**), and tube sample build orientation definitions (**b**).

An AM250 selective laser melting (SLM) machine (Renishaw, Wotton-under-Edge, Gloucestershire, UK) within the class of LM-PBF systems equipped with a pulse modulated Ytterbium fiber laser was used to fabricate the tube samples in the IN718 alloy using three different processing parameters. The build space for parts on the AM250 is (mm) 250 length × 250 width × 300 height. The nominal dimensions of each tube sample (mm) are outer diameter 60, wall thickness 2, and height 55. The IN718-0405 powder material supplied by Renishaw for additive manufacturing was spherical, with the particle diameter between 15 and 45 µm and the specification for composition displayed in Table 1. For tube group A, the supplier recommended that VED settings were used. For each powder layer, the VED for an area scan was 74.1 J/mm^3^, and for the border scan was 185 J/mm^3^. For tube groups D and E, the VED was increased respectively for the layer area scan to 139 and 167 J/mm^3^ and the border scan to 486 and 583 J/mm^3^, respectively. The laser spot of the AM250 scans each layer at an angle of 67° with respect to the previous one. Meander laser scanning was used for each layer and the beam compensation was set to 60 μm. Common process parameters for each sample (μm) was D = 30, H = 90, and spot size 70. The build orientation and corresponding process parameters are displayed in Figure 2 and Table 1. The recorded build time was 47 h and 30 min and the mass of powder used was 0.83 kg. The temperature of the elevator supporting the build plate was maintained at 170 °C through the build process. The argon purge pressure in the build chamber maintained an atmosphere with oxygen content <1000 ppm (0.1%) in accordance with [34]. 

All the tubes were stress relieved while still attached to the build plate at 982 °C for 1 h followed by a slow furnace cool (Renishaw, Wotton-under-Edge, Gloucestershire, UK). Afterwards, the tube samples were wire cut from the build plate using CNC electrical discharge machining (GF Machining Solutions, Coventry, UK). 

The AM tube samples were neither subjected to HIP nor to strength hardening heat treatments. The microhardness testing of the three tube samples was carried out on the build axis plane, which is perpendicular to the build direction (Figure 2b). The range of the major diagonal indents for 10 microhardness readings in each tube sample was between 30 and 36 μm with an average hardness for A, D, and E, respectively 299, 314, and 293 HV_0.2_. The macroscopic porosity was determined on the tube build axis plane (Figure 2b) at three different positions for each AM tube sample. Porosity measurements were performed using a Keyence VHX6000 digital light microscope (Keyence International, Osaka, Japan), together with a Keyence Z1000UR lens at a low magnification of 500× for the measurement of the pore diameter (PD) typically above 5 μm. The region covered for each image to measure porosity was approximately 5 mm^2^. Table 2 displays the porosity measurements obtained at three different positions for each AM tube sample. A Phenom ProX/CeB6 scanning electron microscope (SEM) (Thermo Fisher Scientific Inc., Eindhoven, The Netherlands) with an acceleration voltage at 15 kV was used to detect the smaller pores. 

The compositional elements of the extruded tubes and LM-PBF samples are displayed in Table 3. Inductively coupled plasma and optical emission spectrometry (ICP-OES) performed by (Element Materials Technology, Rotherham, UK) was used to confirm the chemical composition of the alloy produced by the LM-PBF. The nominal dimensions of the extruded IN718 tube was (mm) 50 outer diameter, 2 wall thickness, and 55 height, with a macro hardness 64 HRC.

### 2.2. Machining Setup, Process Parameters, and Cutting Tool

**Figure 3:**
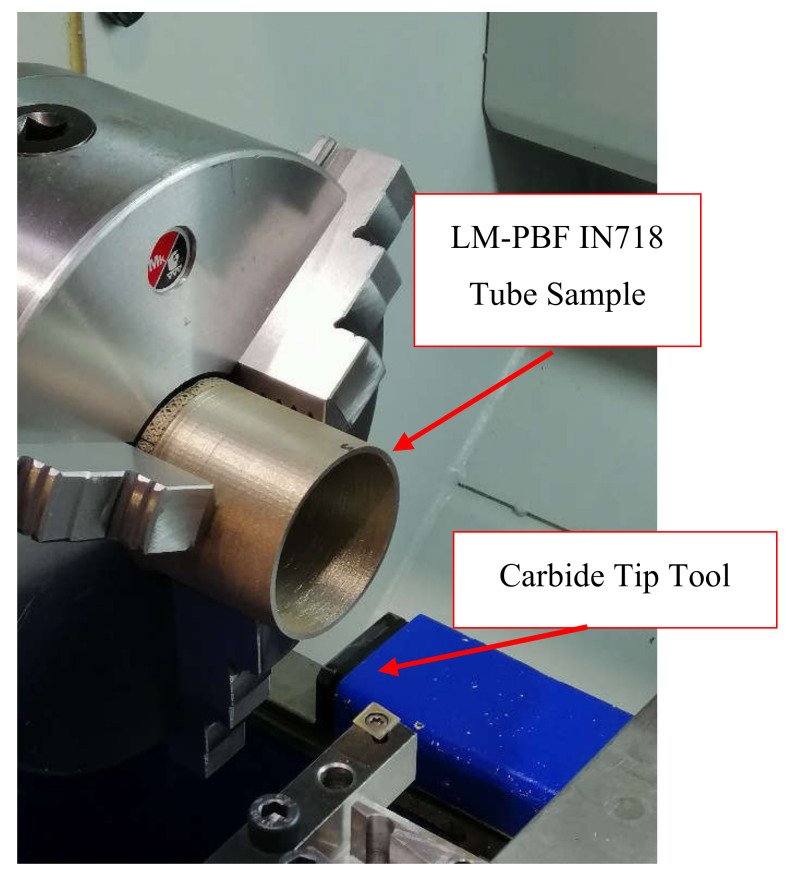
Experimental setup for machining.

The orthogonal turning tests were performed using a Pinacho SmartTurn 6/165 lathe (Pinacho, Huesca, Spain) with the setup displayed in Figure 3. The cutting and feed forces were measured at 1000 Hz simultaneously using a multi-component Kistler dynamometer type 9257B (Kistler, Winterthur, Switzerland). The instrumentation used was an 8-channel amplifier Kistler 5070 (Kistler, Winterthur, Switzerland) and data acquisition card Ni USB 6361.

A SECO TS200 carbide tipped tool incorporating a TiAl/TiAlN coating (Seco, Fagersta, Sweden) was used for light cut machining. The carbide tip geometry has an 80° tip angle, 0.4 mm tip radius, 16° rake angle, 7° relief angle, and a cutting edge radius of 25 µm. In earlier studies, it was demonstrated [14,35] that the tool life in the conditions to be studied for the IN718 alloy was 30 min. The duration of each test cut will be typically 2 s after which a new tip edge will be used for the next test. Therefore, it is reasonable to assume that the tool wear will be negligible, enabling multiple sequential tests to be performed under the same conditions and minimizing noise factors associated with the process. 

The turning tests were performed at three levels with Vc (m/min) at 60, 90, and 120, f (mm/rev) 0.1, WoC (mm) 2, and without the coolant. Each tube specimen (manufactured additively and extruded) were subjected to cutting tests five times (at different locations) for 2 s. At the end of each test cut, the tool was verified to ensure there were no obvious signs of wear.

## 3. Results and Discussion

**Figure 4:**
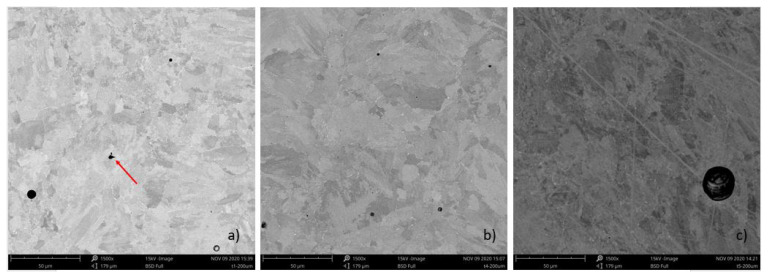
SEM images of spherical porosity in tube samples: (**a**) Tube A, (**b**) Tube D, (**c**) Tube E.

SEM images of porosity on the build axis plane (in the tool feed motion direction) of tube samples A, D, and E are displayed in Figure 4. Tube samples A, D, and E display the spherical porosity although tube sample A with the lowest VED at 74.1 J/mm^3^, displays a LoF defect identified by the red arrow. In the Figure 4 images, the pore diameters displayed for sample A and D are below 10 μm, while for sample E with the highest VED at 167 J/mm^3^ it is approximately 25 μm. In all the sample images examined, the maximum pore diameter (μm) did not exceed 25, 51, and 94, respectively in tube samples A, D, and E. In each of the AM tube samples, the spherical pores on the build axis plane appear randomly dispersed over the tube thickness. The pore diameter also appeared to vary randomly up to the maximum diameter determined for each tube group. The variability in the measured porosity (determined by dividing the standard deviation by the average value) across tube samples A, D, and E is considered high respectively at 0.22, 0.46, and 0.13. However, the trend observed for the increasing porosity in the AM produced IN718 alloy with a higher laser energy density has been reported in the literature.

The LM-PBF samples produced were within the Renishaw specification composition elements [34]. However, it is noted that the percentage mass faction Co content in the AM produced samples (0.09) is slightly smaller than the extruded tube (0.4). Both values are well below the maximum limit (1%) defined by the IN718 alloy specification for composition elements. A slightly higher Co content may increase the strength of the alloy by a small margin following solution annealing and precipitation age hardening heat treatments, but none of the samples were subject to strength hardening heat treatments. The Si content in the extruded tube was higher than that of the AM tubes. The extruded sample has been homogenised and during the latter process, the Si from the laves phase dissolved in the matrix of the alloy accounting for the higher Si content. However, this difference is unlikely to affect the mechanical properties [36]. 

Although the grain structure of the tube samples was not examined in this paper, the microhardness measurements of tube samples A, D, and E on the build axis plane was determined, respectively at 299, 314, and 293 HV_0.2_. The AM tube samples A and E fall within the specification of the alloy [34] which identifies 277 and 302 HV_0.5_, respectively for vertical surfaces of as-built samples without the hardening heat treatment. The exception was sample D with the hardness at 314 HV_0.2_. The microhardness measurements are broadly consistent, but on the upper limit of the alloy specification in the as-fabricated condition. All the tube samples were machined using the same configuration with the tool feed motion applied perpendicular to the build axis plane of the AM tube samples. 

The evolution of the cutting (F_c_) and feed forces (F_f_) with time has been recorded during each turning test for the IN718 extruded and AM produced tubes. The mean value and the variation obtained for F_c_ and F_f_ will be examined using the coefficient of variation (CoV). Other measures to study the results include the coefficient of friction (μ).

**Figure 5:**
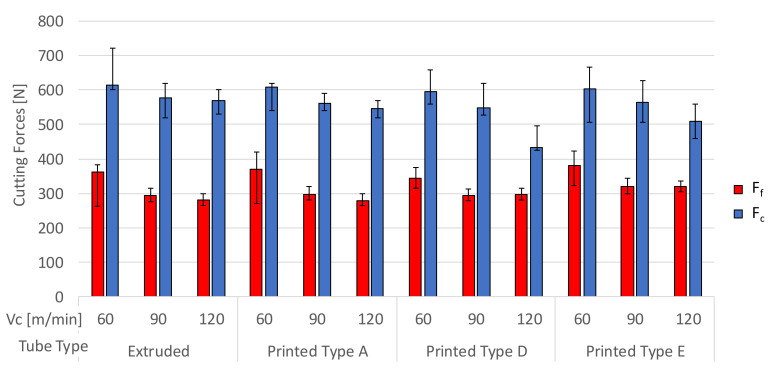
Average cutting forces (F_c_ and F_f_) for the extruded and AM tube samples A, D, and E with a variation in Vc.

The mean value of F_c_ and F_f_ obtained for each cutting speed (Vc) for the extruded and AM tube samples with varying porosity is displayed in Figure 5. The error bar shown for each result identifies the range obtained from the five samples tested for each material and machining condition. All the tube samples display the same value for F_c_ at 60 m/min and there is no discernible difference in F_c_ and F_f_ for the extruded and AM tube sample A (with the lowest porosity at 0.09%) across the Vc range. With the increasing Vc from 60 to 120 m/min, a reduction in F_c_ is observed and more appreciably for tube samples D and E that contain a higher porosity. The F_f_ decreases with the increasing Vc but stabilizes at 90 m/min across all the tube samples. The trends suggest that the thermal softening behaviour in cutting the IN718 alloy at a higher Vc known to be present in the extruded sample in the range examined [15], is also present in the AM samples with varying levels of porosity.

**Figure 6:**
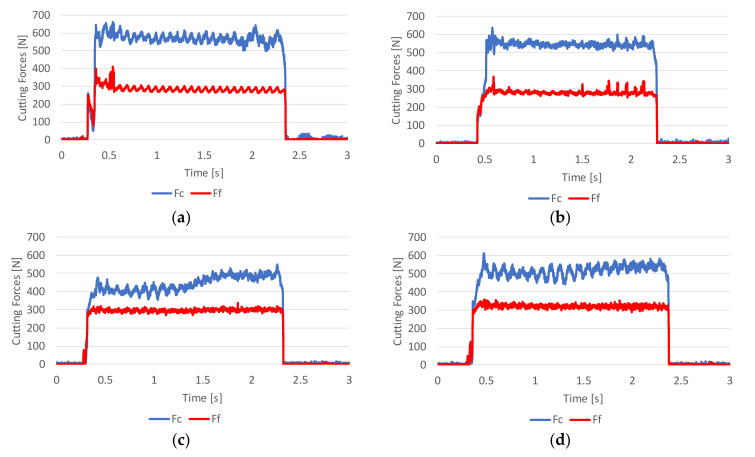
Cutting forces evolution (F_c_ in blue, F_f_ in red) for the tested tubes at a cutting speed of 120 m/min: (**a**) Extruded tube, (**b**) AM tube A, (**c**) AM tube D, (**d**) AM tube E.

Examining the evolution of F_c_ and F_f_ forces with time in Figure 6 for Vc at 120 m/min, while F_f_ stabilizes at a constant value across all the results, F_c_ stabilizes only for the extruded and AM sample A with the lowest porosity. For the AM tubes with increasing porosity, respectively D and E, F_c_ increases with the duration of the cut but more noticeably for AM sample D.

**Figure 7:**
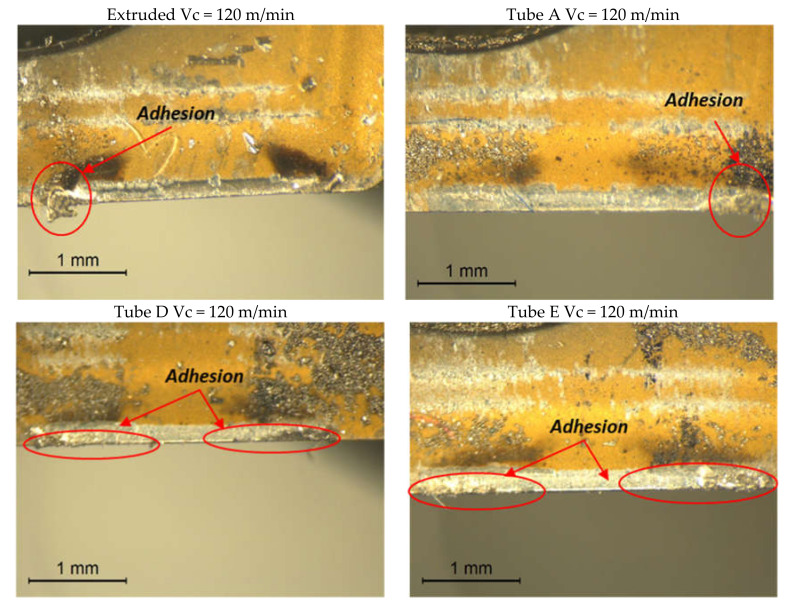
Rake face of the cutting tool tip used for machining the extruded and the AM tubes with Vc at 120 m/min.

F_c_ increases with time for the AM tubes D and E at 120 m/min, since there is an increasing tendency of the workpiece alloy to adhere to the rake face of the cutting tool tip as a built-up-edge (BUE), despite the short duration of the test (2 s). Figure 7 displays the rake face of the cutting tool tip with Vc at 120 m/min for each tube sample in which BUE can be observed. The wear that develops is a notch-type wear and the rate of BUE increases for AM tube samples D and E, which display a greater adhesion, and this was also observed with Vc at 90 m/min [14,35] for the wrought IN718 alloy.

An average measure for the coefficient of friction (µ) is determined by dividing the feed force (F_f_) by the cutting force (F_c_).

µ = F_f_/F_c_ (2)

Equation (2) identifies that µ is proportional to F_f_/F_c_. The calibration of Equation (2) would require a tool tip cutting edge geometry such as the rake angle and edge radius. In this paper, the tool tip geometry is not a variable, and Equation (2) is used for the comparative analysis of all the results generated.

**Figure 8:**
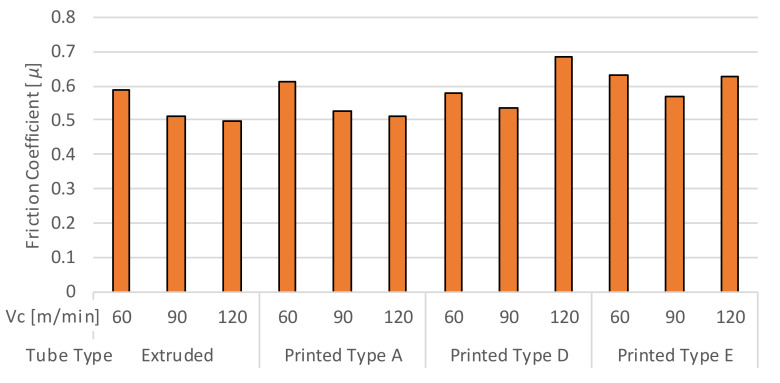
Mean coefficient of friction for the extruded and AM tube samples.

The effect of Vc on µ is displayed in Figure 8 below. As Vc increases from 60 to 90 m/min, µ reduces for all the tube samples, suggesting an improved lubricity between the tool and workpiece that could be attributed to thermal softening in the plastic deformation zone that precedes chip formation. For the extruded and AM tube A, µ stabilizes at 90 m/min. At higher Vc of 120 m/min, µ increases for tube samples D and E containing a higher porosity. For tube sample E, with the highest porosity, μ across the Vc range is typically higher than all the other samples. This suggests a propensity to BUE formation with the increasing porosity across the Vc range. Examining Figure 5, at the highest Vc, the tube samples with higher porosity (AM Tube E) delivered higher F_f_. Furthermore, F_f_ was the same in the Vc range from 90 to 120 m/min. A similar thermal softening of a workpiece was previously observed by Samad Nadimi Bavil Oliaei [37], where they found that the presence of a built-up edge affects the thrust forces more than the cutting forces.

The normalized measure of variation was computed using the coefficient of variation (CoV), which is a function of the standard deviation [σ]. It is defined by the expression:

CoV = 100 (%) × σ/(Mean value of the F_c_) (3)

**Figure 9:**
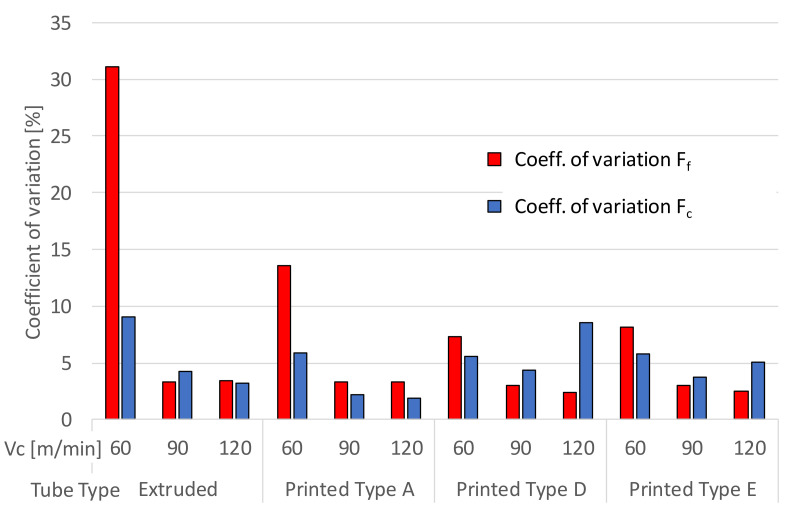
Coefficient of variation (F_c_ and F_f_), for the extruded and AM tube samples.

Figure 9 displays the CoV across the results obtained. All the results for F_c_ are below 10% and the AM tube sample A displays the lowest CoV. The same trend for decreasing CoV with the increasing Vc is observed for the extruded tube and AM tube sample A. However, CoV for F_c_ obtained for the AM tube samples D and E increases at the highest Vc of 120 m/min. 

**Figure 10:**
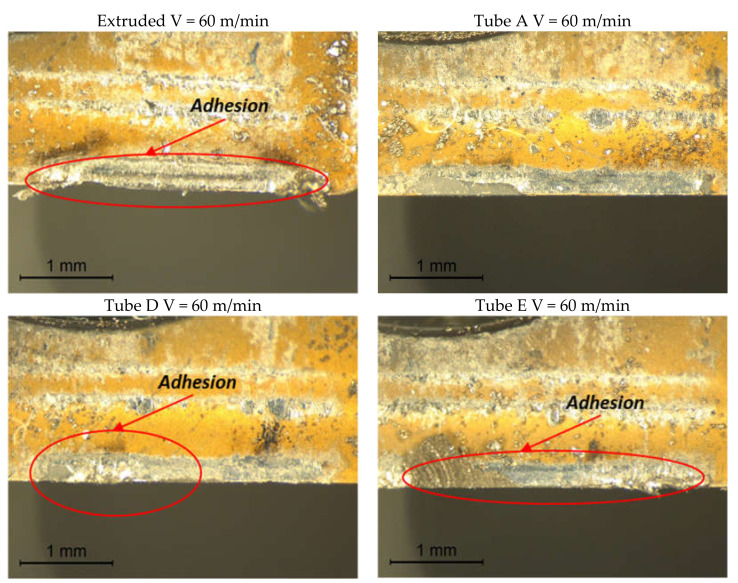
Rake face of the tool tip used for machining the extruded and the AM tubes with Vc at 60 m/min.

The CoV for F_f_ at 60 m/min for the extruded and AM tube sample A were the highest exceeding 30% and 13%, respectively. The high CoV for F_f_ was obtained with Vc at 60 m/min and was attributed to resonance in the feed motion encountered with Vc at 60 m/min for the experimental arrangement. With the vibration present in the cutting process, BUE readily formed on the tool tip rake face as shown in Figure 10.

Based on the CoV obtained for the cutting (F_c_) and feed forces (F_f_), the stability of the process is improved with the increasing Vc for the extruded and AM tube sample A (least porosity). The lower CoV obtained for F_f_ at 60 m/min for tube samples D and E with the higher porosity may affect the cutting process in several ways. For example, the material flow under the tool which directly influences the F_f_ and possibly slightly improved damped properties of the alloy.

The findings in this paper suggest that the increasing levels of microporosity in the workpiece promote a tendency of the workpiece material to adhere to the rake surface of the tool tip. The chip with microporosity, which is removed from the workpiece surface promotes instability in the main cutting direction (F_c_). The higher rate of adhesion on the tool tip increases the friction, leading to a higher temperature in the secondary deformation zone (interface relief surface of the tool-workpiece), which softens the material to reduce ploughing and stabilise the force in the feed direction.

## 4. Conclusions

In this paper, the machinability of AM tube samples produced by laser melting powder bed fusion in the INCONEL718 alloy with varying porosity in the microstructure, was compared with the extruded wrought tube product in the same alloy under light cut conditions to simulate a finishing process. The orthogonal turning of the tube samples with a 2 mm wall thickness was performed under dry cut conditions, while varying the cutting speed in the range of 60, 90, and 120 m/min with the feed 0.1 mm/rev, each over a 2 s cut duration. It was found that the mean cutting force and coefficient of friction obtained for the AM tube with the lowest porosity were consistent in trend and magnitude with the extruded wrought tube product across the range of the cutting speeds examined, both measures decreasing with the cutting speed. By comparison, the AM tube samples with a higher porosity displayed comparatively lower cutting forces at a higher cutting speed. However, the coefficient of friction generally increased, especially at the highest cutting speed and this is attributed to the higher rate of BUE formation on the tool rake face despite the short cut duration. 

## Figures and Tables

**Table 1:** AM processing parameters used for the laser melting powder bed fusion (LM-PBF) of tube samples A, D, and E.

LM-PBF Tube ID	Units	A		D		E	
		Fill	Border	Fill	Border	Fill	Border
Laser power	W	200		175		175	
Exposure time	µs	70	50	150	150	180	180
Point distance	µm	70	20	70	20	70	20
Scan speed	mm/s	1000	400	467	133	389	111
Energy density	J/mm^3^	74.1	185	139	486	167	583

**Table 2:** Porosity measurements of LM-PBF tube samples A, D, and E.

LM-PFF Tube ID	Units	A			D			E		
Sample Number		1	2	3	1	2	3	1	2	3
Porosity	(%)	0.07	0.10	0.09	0.49	0.14	0.28	3.29	4.01	2.98
Average	(%)		0.09			0.30			3.43	
Standard deviation	(%)		0.01			0.14			0.43	
Sigma/Average			0.22			0.46			0.13	

**Table 3:** Chemical composition of the INCONEL718 (IN718) extruded tube, LM-PBF sample, and Renishaw IN718 powder specification [34].

Element (m%)	Al	C	Co	Cr	Cu	Fe	Mn	Mo	Ni	S	Si	Ti
Extruded	0.53	0.053	0.40	18.3	0.05	18.6	0.24	3.04	52.3	<0.002	0.09	1.10
LM-PBF	0.47	0.053	0.09	18.4	0.01	18.0	0.01	3.08	53.7	<0.003	0.03	0.95
Renishaw powder (0405) [34]	0.2 to 0.8	0.02 to 0.05	<1.0	17.0 to 21.0	<0.3	Balance	<0.35	2.8 to 3.3	50.0 to 55.0	<0.015	<0.35	0.65 to 1.15
